# The Shear Stress Determination in Tubular Specimens under Torsion in the Elastic–Plastic Strain Range from the Perspective of Fatigue Analysis

**DOI:** 10.3390/ma13235583

**Published:** 2020-12-07

**Authors:** Jan Seyda, Łukasz Pejkowski, Dariusz Skibicki

**Affiliations:** Faculty of Mechanical Engineering, University of Science and Technology in Bydgoszcz, Kaliskiego 7, 85-796 Bydgoszcz, Poland; jan.seyda@utp.edu.pl (J.S.); dariusz.skibicki@utp.edu.pl (D.S.)

**Keywords:** cyclic torsion, shear stress, tubular specimens, elastic–plastic torsion

## Abstract

The comparison of shear stress determination methods in tubular specimens under torsion is presented in this paper. Four methods were analyzed: purely elastic solutions, purely plastic solutions, the midsection approach, and the Chaboche nonlinear kinematic hardening model. Using experimental data from self-designed and conducted fatigue experiments, an interesting insight on this problem was obtained that is not often tackled in the literature. It was shown that there are differences in determined shear stress values, and their level depends on a few factors. The midsection approach and purely plastic solution gave values of surface shear stress very close to the values obtained using the Chaboche nonlinear kinematic hardening model for high strain levels. The purely elastic solution gave proper results for the low strain ranges, close to the cyclic yield limit. Since none of the methods can be trusted in the full range of loading, an important conclusion from these analyses regards the formulated ranges of their applicability. It was also shown that the calculated values of shear stress and plastic and elastic strain energy density determined on this basis have a strong impact on fatigue life predictions. Finally, the influence of predicted values of shear stresses on the interpretation of cyclic hardening phenomena was also presented.

## 1. Introduction

Usually, fatigue cracks originate on a free surface due to the stress concentration on a micro and macro scale. Given polycrystalline materials, plastic deformation is also higher on a surface as a result of lower constraints from neighboring grains [1]. In the case of elements that are loaded with bending moment or torque, stresses are additionally higher on a surface due to their gradient [2,3]. These types of loading are often studied [4,5], also in an elastic–plastic strain range [6,7]. Considering this, it is clear that the proper analysis of stress and strain on the surface of elements undergoing fatigue loading is an important issue. On the other hand, a compromise between the accuracy and simplicity of the solution is usually desired.

In the present study, the focus is placed on the analysis of shear stress in thin-walled tubular specimens, which are most often used in strain-controlled multiaxial fatigue tests [8,9]. The problem of elastic–plastic torsion has already been studied by other authors. Usually, some advanced mathematical models are made regarding this issue, together with proposals of numerical solutions devoted to the determination of the twist angle or accumulated energy [10,11,12,13,14,15,16]. Other papers concern an application of the Finite Elements Method or the Finite Differences Method to model the torsion of different profiles [17,18,19]. Sometimes, the topic of the study is very narrow, e.g., it concerns only a selected issue related to the yield point, which the authors term the “small corrugations of the elastic–plastic boundary” [20]. Unfortunately, quite often such works do not provide practical solutions and explanations on how to correctly determine the surface shear stress for tubular specimens subjected to cyclic torsion, based on experimental data like torque and shear strain on the surface of the specimen. If any cyclic plasticity models are involved, they assume perfectly elastic or perfectly plastic material behavior. More practical considerations regarding the cyclic torsion of cylindrical specimens have previously been performed by Miller and Chandler [21] and Brown [22].

In fatigue analysis, authors use various approaches to solve the problem of shear stress determination in tubular specimens under torsion. Shamsaei and Fatemi [23] used midsection shear stress values, which were further fitted with a Ramberg–Osgood-type function and extrapolated on the surface. However, this method can be used only for materials that exhibit Masing behavior (the upper branches of hysteresis loops form one common curve) and do not show significant differences in stress history depending on the loading level. Furthermore, the fit and extrapolation procedure must be separately repeated for each loading case. The midsection approach was also used by Socie [24], Zhong et al. [25], Wu et al. [26], Zhang and Jiang [27,28], and in other works by Fatemi and coauthors [29,30]. Dey et al. used an elastic solution, even though they applied relatively high strain values [31]. There are also several papers where the shear stress determination method was not mentioned [32,33,34,35].

In the present paper, three different approaches based on the measured value of torque were applied to determine shear stress in tubular specimens made of PA38-T6 (AW-6060) aluminum alloy subjected to torsion. They were further compared to values calculated using the Chaboche kinematic hardening model. This thorough approach to the problem makes it possible to become aware of differences in shear stress values obtained using various methods and to find their dependence on factors such as specimen size and loading level. The results of this work also give an idea of the order of magnitude of these differences, the impact on the interpretation of cyclic hardening, and on the prediction of fatigue life. Considering these factors, the guidance was provided according to the applicability of these methods. Furthermore, it is worth noting that all analyses were conducted based on a self-designed and conducted experiment. The adopted research methodology of the experiment assumed continuous recording of all signals from the testing system with a high sampling frequency. This enabled accurate calculations and detailed analyses.

## 2. Theoretical Background

### 2.1. Shear Stress Distribution under Elastic–Plastic Torsion

**Figure 1:**
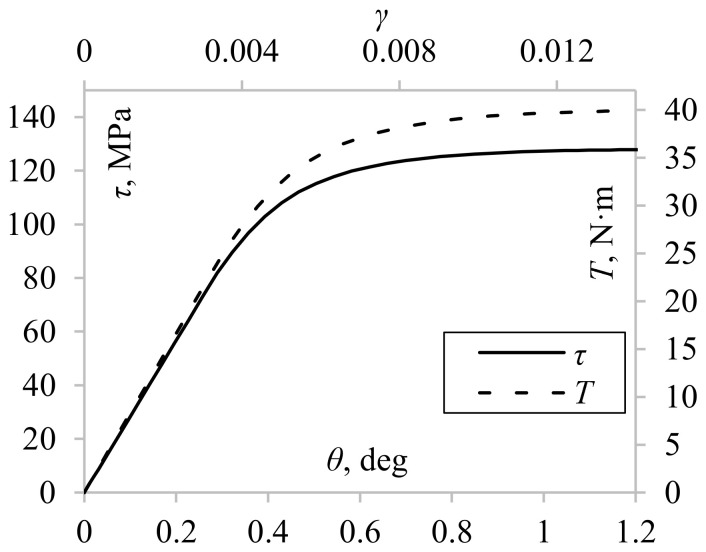
An example of shear-stress–shear-strain (τ-γ) and torque–twist angle (*T*-θ) curves.

**Figure 2:**
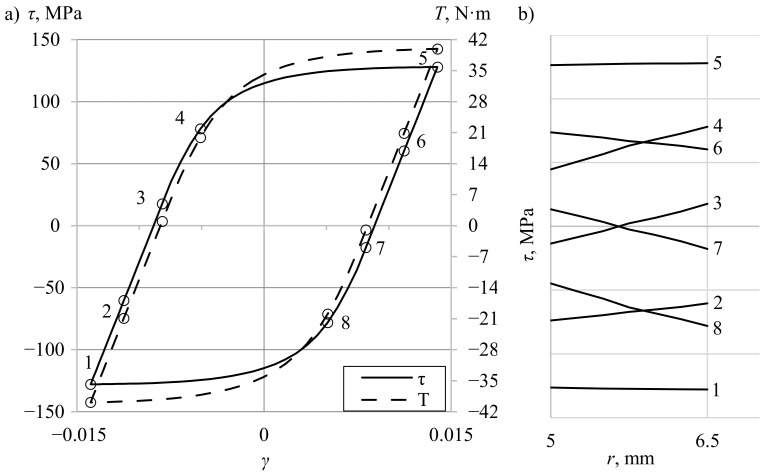
An example of (**a**) shear-stress–shear-strain (τ-γ) and torque–shear-strain (*T*-τ) hysteresis loops and (**b**) shear stress distribution through the wall thickness for 8 selected points.

Nonuniform shear stress distribution occurs in every specimen subjected to torsion if the stress is higher than the yield limit [19]. Such distributions make it dubious to apply simple elastic solutions for accurate determination of shear stress since they assume a linear relationship between stress and strain. Furthermore, several other phenomena influence the shear stress value on the surface of the specimen. For example, the torque can be high enough to induce yielding of the outer layers of the material, but at the same time small enough to remain elastic at internal layers [12]. The boundary between elastic and elastic–plastic deformation also moves along the radius during the cyclic loading and unloading processes [21]. The torque, which is a measured quantity, depends on the stresses acting in the whole cross-section of the specimen; thus, the stress–strain and torque–twist angle relationship differ (Figure 1). These relationships become even more complicated when considering cyclic loadings [36]. When twisting right (conventionally) and subsequent unloading, the stress in the outer layer is not yet zero, while the stress in the internal layer exceeds zero, so it is already compressed (stressed left) [18] (Figure 2). Therefore, when the torque being measured equals zero, the value of shear stress in the external layer differs from zero (points 3 and 7). This influences the shape of the hysteresis loop and the calculated plastic strain energy density. If the material hardens or softens cyclically, the amount of hardening/softening depending on strain level varies for different layers (in the radial direction) of the specimen. This hardening or softening is difficult to consider during the analysis of data that is only recorded from the testing system. All the abovementioned phenomena depend further on the level of loading, on internal and external diameters of the specimen, and on the specimen’s thickness.

### 2.2. Selected Methods of the Shear Stress Determination in Case of Tubular Specimens

The first of the considered approaches for determining shear stress in tubular specimens is based on a purely elastic solution. The relationship between torque *T* and shear stress τ is given by [37]

(1) $$T=\int_{A}\tau \left(r\right)rdA=2\pi \int_{r}\tau \left(r\right)rdr=2\pi \int_{r_{i}}^{r_{o}}\tau \left(r\right)\cdot r^{2}dr,$$

where $r_{i}$ and $r_{o}$ are internal and outer radii of the specimen, respectively. By the assumption that the shear stress is a linear function of radius, τ(r)=ar, where *a* is the slope of function, after integration and substitution of *a* by $\tau_{o}$, we get

(2) $$T=\frac{\pi \tau_{o}\left(r_{o}^{4}-r_{i}^{4}\right)}{2}.$$

**Figure 3:**
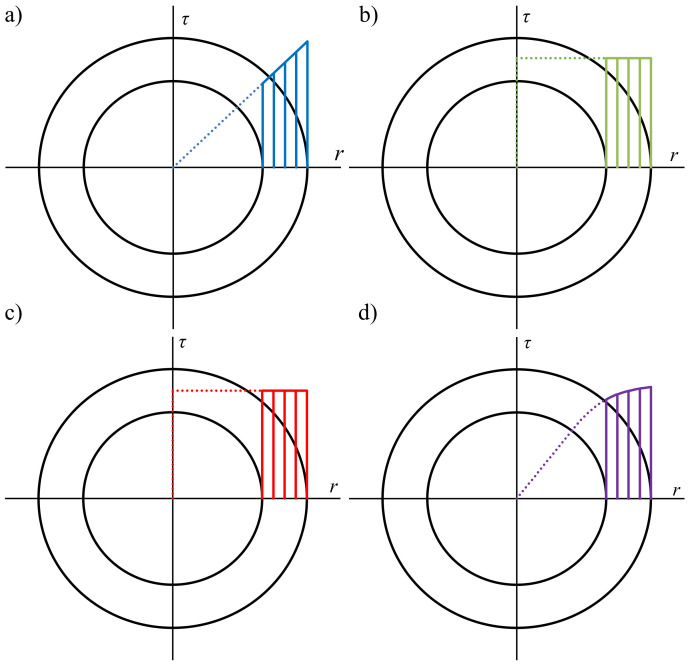
Shear stress distribution along the radius of a tubular specimen for (**a**) elastic solution, (**b**) midsection approach, (**c**) plastic solution, and (**d**) Chaboche model.

The shear stress on the surface is then given by

(3) $$\tau_{o}=\frac{T}{J_{0}}r_{o}=\frac{2r_{o}T}{\pi (r_{o}^{4}-r_{i}^{4})},$$

where $J_{0}$ is the polar moment of inertia. The resulting distribution of shear stress along the radial direction is presented in Figure 3a.

The second method is recommended by the ASTM standard [57] for the low-cycle fatigue regime. It is assumed that the shear stress distribution is uniform through the wall thickness (Figure 3b) and is equal to the midsection value $\tau_{m}$. The torque is given by the following simplified Equation (1):

(4) $$T=\tau_{m}r_{m}A,$$

and $\tau_{m}$ by

(5) $$\tau_{m}=\frac{T}{Ar_{m}}=\frac{16T}{\pi \left(d_{o}^{2}-d_{i}^{2}\right)\left(d_{o}+d_{i}\right)},$$

where $r_{m}$ is the mean radius. The standard also specifies the criterion for a thin wall, for which the shear stress variation in the radial direction is negligible. Specimens that do not meet the criterion of a thin wall are examined in the work of Brown [22].

The next approach is based on a purely plastic solution. The assumption is the same as in the case of the midsection method (Figure 3c), and the relationship between shear stress and the radius of the specimen is given by

(6) τ(r)=const.

After substituting (6) into (1) and integration, the following formula is obtained:

(7) $$T=\frac{2}{3}\pi \tau \left(r_{o}^{3}-r_{i}^{3}\right),$$

and after conversion, the shear stress on the surface is then given by

(8) $$\tau_{o}=\frac{3T}{2\pi (r_{o}^{3}-r_{i}^{3})}.$$

The shear stress distributions and values obtained by the previously presented approaches were compared to the values calculated using the Chaboche nonlinear kinematic hardening model [38,39]. The set of applied equations is listed below. The yield surface according to Huber–Mises yield criterion describes the formula:

(9) $$f=\sqrt{\frac{3}{2}\left(s-\alpha \right):\left(s-\alpha \right)}-R,$$

where *s* is a deviatoric stress tensor, *R* is cyclic yield stress, and α is a backstress tensor given by

(10) $$d\alpha =\sum_{i=1}^{m}\frac{2}{3}C^{\left(i\right)}d\varepsilon^{p}-\gamma^{\left(i\right)}\alpha^{\left(i\right)}dp,$$

where $C^{\left(i\right)}$ and $\gamma^{\left(i\right)}$ (not to be confused with the shear strain) are material parameters and $d\varepsilon^{p}$ is a plastic strain tensor rate, given by the associated plastic flow rule:

(11) $$d\varepsilon^{p}=\frac{3}{2}\frac{ds:n}{H}n,$$

where *n* is an outward unit tensor determining the direction normal to the yield surface:

(12) $$n=\frac{\frac{\partial f}{\partial s}}{\sqrt{\frac{\partial f}{\partial s}:\frac{\partial f}{\partial s}}}.$$

*H* is a plastic hardening modulus and dp is the accumulated equivalent plastic strain rate:

(13) $$dp=\sqrt{\frac{2}{3}d\varepsilon^{p}:d\varepsilon^{p}}.$$

The value of *H* can be determined from the yield surface consistency condition:

(14) $$\frac{\partial f}{\partial s}:ds+\frac{\partial f}{\partial \alpha }:d\alpha =0.$$

Using the Chaboche model, a nonlinear distribution of the shear stress along the specimen’s radius is achieved (Figure 3d).

### 2.3. Influence of Shear Stress on Fatigue Life Prediction

For fatigue of materials, the ability to determine the correct shear stress value is crucial. The most popular multiaxial fatigue damage parameters, like Fatemi–Socie [40], generalized Smith–Watson–Topper [24,41], Łagoda–Macha [42], Ince–Glinka [43], or Garud [44], depend directly or indirectly on the determined value of this stress. Such a fatigue damage parameter is proposed by Gołoś and Ellyin [45,46]:

(15) $$\frac{\Delta W^{p}}{\rho }+\Delta W^{e+},$$

where $\Delta W^{p}$ and $\Delta W^{e+}$ are total plastic and positive elastic strain energy densities and ρ is a multiaxiality coefficient given by

(16) $$\rho =\left(1+\nu_{eq}\right)\frac{\varepsilon_{1}}{\gamma_{max}},$$

where $\nu_{eq}$ is an equivalent Poisson ratio, $\varepsilon_{1}$ is maximum principal strain, and $\gamma_{max}$ is the maximum shear strain. The Gołoś–Ellyin parameter was further employed in the present work to analyze the influence of the shear stress value on fatigue life prediction in an energy-based approach.

## 3. Materials and Methods

**Figure 4:**
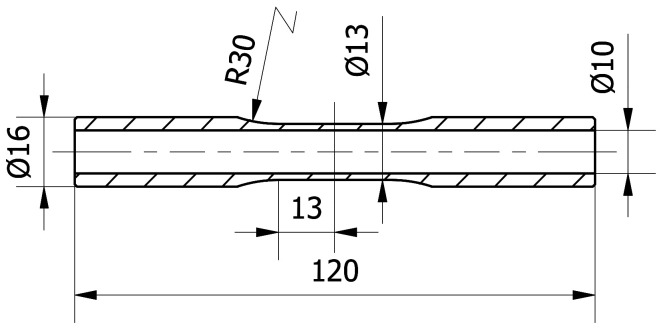
Dimensions of PA38-T6 specimens, given in millimeters.

The experimental data used in this work comes from a broader research program [47,48]. The present work considers previous research regarding the cyclic torsion and axial loading of tubular specimens made of PA38-T6 (AW-6060) aluminum alloy. The chemical composition of this material is given in Table 1. Basic mechanical properties, Young’s modulus *E*, 0.2% offset yield stress $\sigma_{y02}$, ultimate tensile strength $\sigma_{u}$ and corresponding strain $\varepsilon_{\sigma_{u}}$, elastic Poisson ratio $\nu_{e}$, cyclic strength coefficient $K^{\prime }$, and cyclic strain hardening exponent $n^{\prime }$ are given in Table 2. The dimensions of the specimens are presented in Figure 4. All fatigue tests were performed on an Instron 8874 servohydraulic axial/torsional testing system. Axial and shear strains were measured and controlled using an Epsilon 3550 biaxial extensometer, and the axial force and torque were measured by the system’s load cell. For further details of the experiment, the reader is referred to [47,48].

The values of the Chaboche model material parameters were determined by using the Interior-Point optimization algorithm to find the best fit of the plastic strain–stress curve for the experimental data recorded for axial loading at midlife [49]. The value of m=3 decomposition parts of the backstress tensor was found to be optimal. Determined material parameters are given in Table 3. All the calculations were further performed analytically, using own-developed Matlab code, employing the tensor algebra. The strain tensor was used as a controlled quantity and the stress tensor increments were determined for the corresponding strain tensor increments.

**Figure 5:**
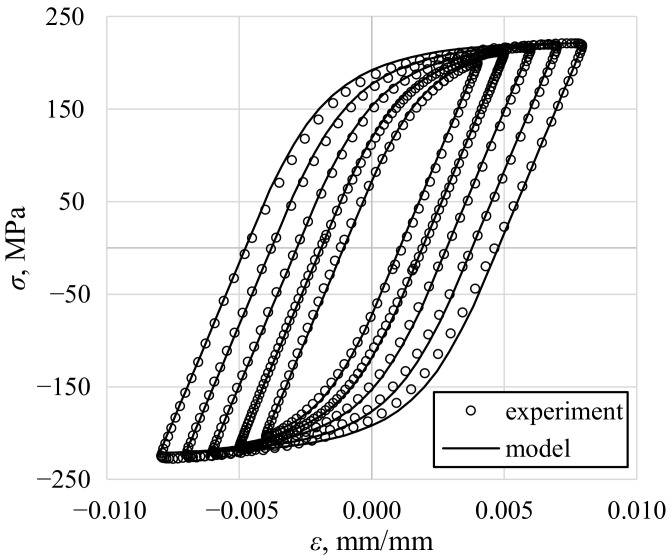
A comparison of experimental and calculated cyclic midlife axial stress–strain response of PA38-T6 aluminum alloy.

The results of the cyclic stress–strain response calculations for fully reversed axial straining are shown in Figure 5. The Chaboche model with correctly determined parameters resulted in accurate modeling of the stress response for several strain amplitudes.

## 4. Results

### 4.1. Shear Stress Distribution Comparison

**Figure 6:**
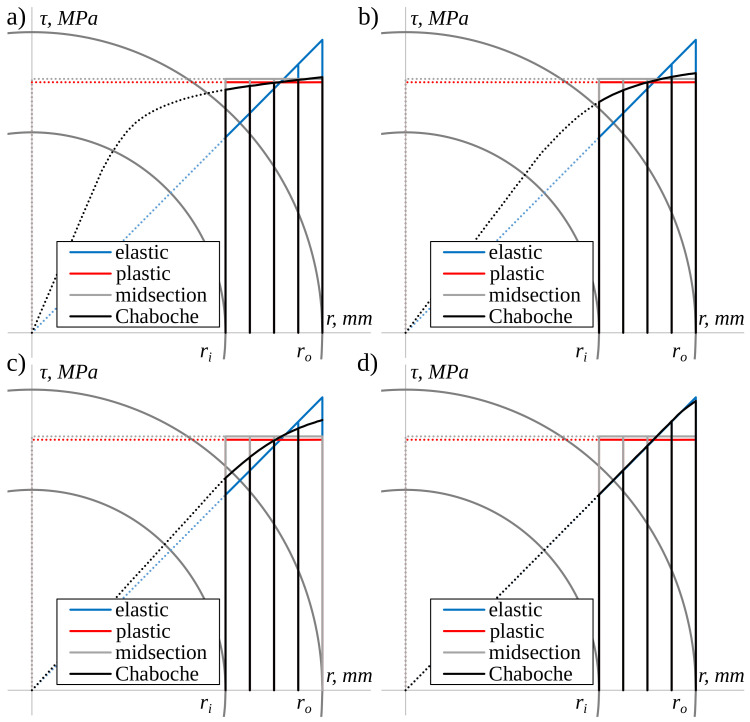
A comparison of the shear stress distribution for the specimen’s theoretical, oversized thickness for (**a**) γ=0.0139, (**b**) γ=0.0069, (**c**) γ=0.0052, (**d**) γ=0.0035.

The shear stress distributions along the radial direction, according to the different shear strain levels determined by the aforementioned approaches, are shown in Figure 6. For this purpose, twice the theoretical thickness of the real specimen (Figure 4) was assumed to best show the differences in shear stress distribution. In the first step, the shear stress distribution for a given shear strain level on the surface was calculated using the Chaboche model. Based on this distribution, the torque was calculated using Equation (1), and then the shear stress distribution was determined using other approaches for the same torque value. This procedure was applied since, in the case of the Chaboche model, the shear stress value is calculated based on the shear strain. With other methods, the shear stress value is determined based on torque. It should be noted that the most important quantity is the shear stress on the specimen’s surface. In reality, it falls between the values resulting from purely elastic and purely plastic solutions.

Based on the similarity between experimental values of stress under axial loading and values calculated using the Chaboche model (Figure 5), it was assumed that when torsion occurs, the shear stress determined using the Chaboche model is closer to the actual values than when it is obtained using other selected approaches. It was then concluded that for high levels of plastic strain, the midsection approach and the purely plastic solution reflect the real values of the shear stress quite well (Figure 6a,b). However, as the strain level decreases and approaches the purely elastic state of deformation, the elastic solution gives better results (Figure 6c,d). Generally, the elastic solution overestimates shear stress with higher strain levels. On the contrary, the midsection approach and the purely plastic solution underestimate shear stress. The underestimation is very small for high levels of strain (Figure 6a,b), while it is higher for small strain levels (Figure 6c,d).

### 4.2. Application of the Selected Methods to Experimental Data Analysis

**Figure 7:**
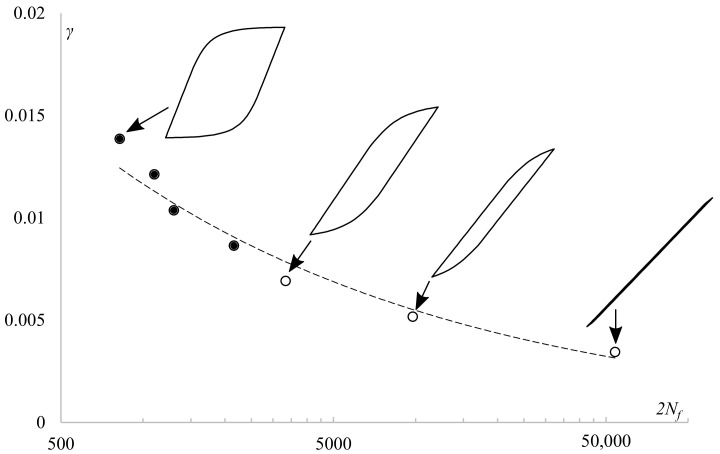
Shear strain amplitudes vs. fatigue life given in reversals for fully reversed torsion of PA38-T6 tubular specimens with midlife hysteresis loops presenting plastic strain level for selected levels of total strain.

In Figure 7, experimental fatigue life was presented against applied shear strain amplitudes. Results were fitted with a power curve. Shear stress was calculated using the midsection method for the purpose of the presentation only. For four selected levels of strain, midlife hysteresis loops were presented to show the plastic-to-total strain ratio with respect to loading level. The applied loading ranges resulted in various stress–strain responses, from almost perfectly elastic to having a high share of plastic strain.

**Figure 8:**
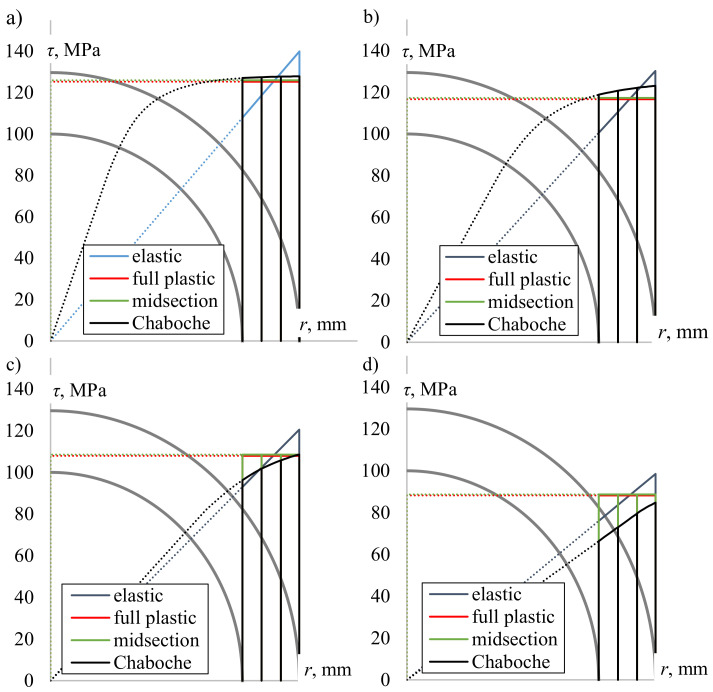
A comparison of the shear stress distribution obtained using selected methods for (**a**) γ=0.0139, (**b**) γ=0.0069, (**c**) γ=0.0052, (**d**) γ=0.0035.

Figure 8 presents the shear stress distribution along the specimen radius obtained using the selected methods for different shear strain levels. The difference compared to the data shown in Figure 6 is that in the case of elastic, plastic, and midsection solutions, the shear stress was calculated directly from the torque measured during the fatigue tests using a load cell. The actual specimens’ diameters were considered as well. Using the Chaboche model, the shear stress was calculated based on the measured shear strain, regardless of the real torque value. It can be seen that for the highest level of loading, i.e., γ=0.0139 (Figure 8a), the surface shear stress determined from torque using the midsection and a perfectly plastic solution was very close to the surface shear stress calculated using the plasticity model. The elastic solution gave higher values. The same observation was made for other relatively high levels of loading, namely, γ=0.0121,0.0104, and 0.0087 (black dots in Figure 7). A noticeable discrepancy between the results obtained from the plasticity model, midsection and the plastic solution began from γ=0.0069 (Figure 8b). For that level of shear strain amplitude, the surface shear stresses determined using the Chaboche model were higher than those calculated using the midsection and plastic methods. Similarly, so was the stress obtained from using the elastic solution. In the case of γ=0.0052, the surface shear stress calculated from the plasticity model, midsection, and plastic solution was nearly the same (Figure 8c). However, the whole shear stress distribution curve based on the Chaboche model is below the midsection and plastic solution. Again, the elastic approach gave higher values. For the lowest examined level of loading (γ=0.0035), the surface shear stress from the midsection and purely plastic solution is considerably higher than the stress calculated by the Chaboche model (Figure 8d).

In general, it is worth noting that if the shear stress distribution curve resulting from the application of the Chaboche model lies above the midsection and plastic values (Figure 8a,b), the experimental torque value was lower than expected from the plasticity model and vice versa. The purely elastic approach always resulted in surface shear stress higher than stress obtained using other applied methods, which seems to be obvious. Although the PA38-T6 aluminum alloy was found to be isotropic, quite cyclically stable, and to follow the Masing behavior, the application of the plasticity model did not offer a significantly better solution than other, much simpler methods. Stress evolution during the experiments can be given here as an explanation.

**Figure 9:**
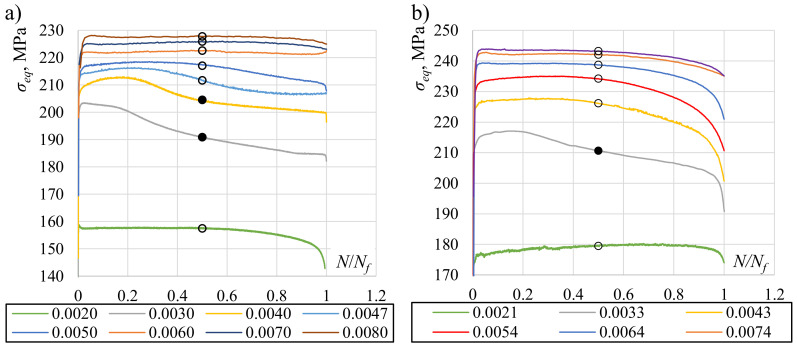
Huber–Mises-equivalent stress histories for (**a**) axial and (**b**) torsional loading, calculated for midsection values; circles indicate midlife values.

**Figure 10:**
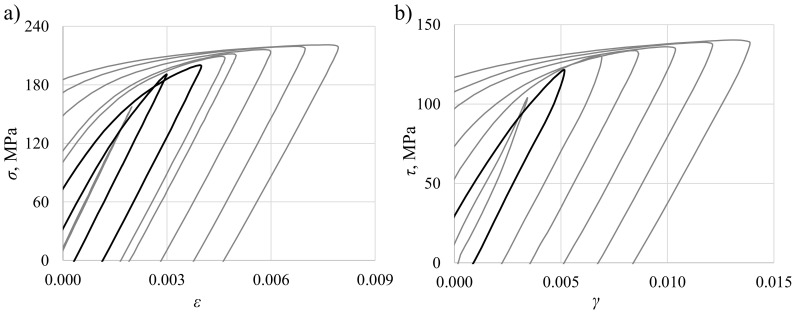
Right-upper quarters of hysteresis loops for (**a**) axial and (**b**) torsional loading; for torsion, the midsection stresses are presented.

In Figure 9, the Huber–Mises midsection equivalent stress histories are presented. Stresses from the beginning of the test to midlife were more stable for high strain amplitudes. The least stability can be seen in the loading levels that result in stress values close to the cyclic yield stress (the curves with black dots). The consequence of this instability is differences in midlife stress values for low levels of loading, as shown in Figure 10 (black hysteresis loops). Similar behavior was also found by other authors [50,51]. A much more sophisticated and complicated cyclic plasticity model would have to be used to describe this phenomenon, which would make its application less practical.

**Figure 11:**
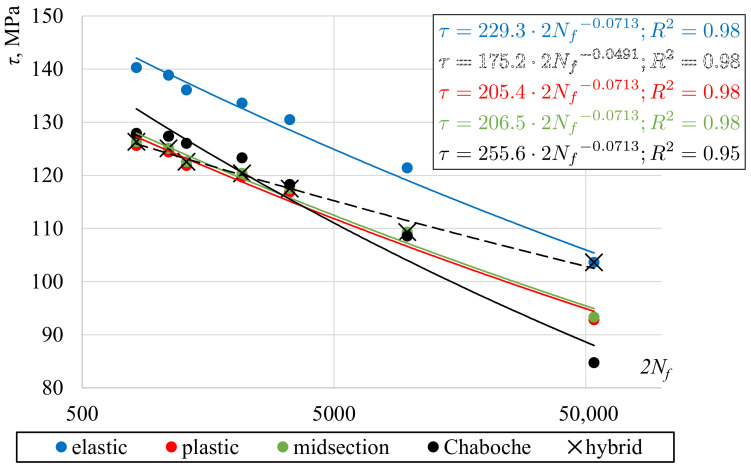
Shear stress vs. fatigue life curves for different shear stress determination methods.

Two quantities often used in fatigue analysis were further evaluated and compared, based on selected approaches to shear stress determination. The first was the maximum surface shear stress. The best-fit power-law shear-stress–fatigue life curves are presented in Figure 11. The curves for the midsection approach and perfectly plastic solution are almost identical. The data points for the Chaboche model are also very close, except for the lowest level of loading. In the case of an elastic solution, the curve is located significantly above the others. If only stress values are considered, about a 10% difference exists between plastic or midsection and elastic solutions. However, due to quite small slopes of the curves, that difference results in fatigue lives eight times higher. In the same figure, a ”hybrid“ curve was also presented. It was fitted for midsection value points corresponding to the six highest levels of strain and one elastic value point corresponding to the lowest level of strain, for which an almost perfectly elastic response was observed. Such a curve seems to be the most adequate.

**Figure 12:**
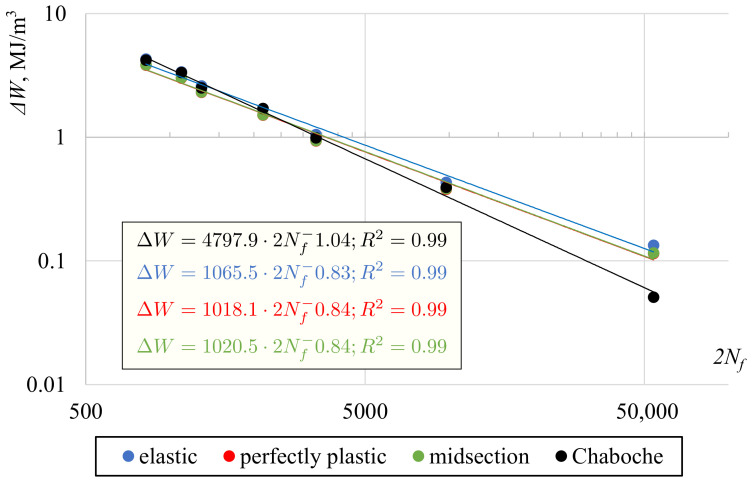
Gołoś–Ellyin energy parameter vs. fatigue lives for different shear stress determination methods.

**Figure 13:**
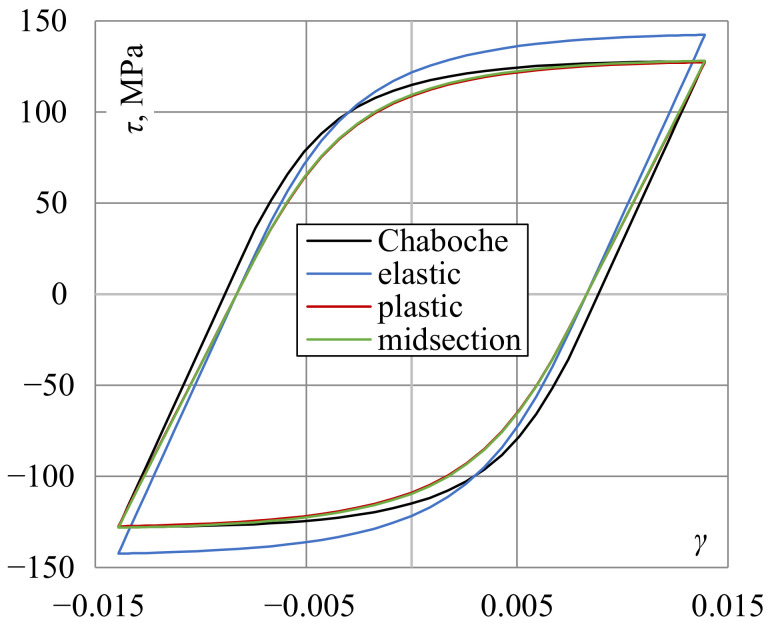
A comparison of shear strain hysteresis loops with stresses determined using different methods for γ=0.0139.

The second selected quantity was a sum of plastic and positive elastic strain energy density, similar to the case of the Gołoś–Ellyin energy parameter (compare (15)). Calculated values of energy are presented in Figure 12. This quantity turned out to be less sensitive for the shear stress determination approach, even for the elastic solution. Differences between the best-fit curves for the midsection approach or the plastic and elastic solutions in terms of fatigue life were approximately 15%. The explanation can be seen in Figure 13, where shear strain hysteresis loops according to different stress determination methods were plotted for γ=0.0139. Compared to the hysteresis loop obtained using the plasticity model, the hysteresis loop based on the elastic solution has stress underestimated for the lower strain range and overestimated for the higher range. However, these differences even each other out and the resulting energies are quite similar.

**Figure 14:**
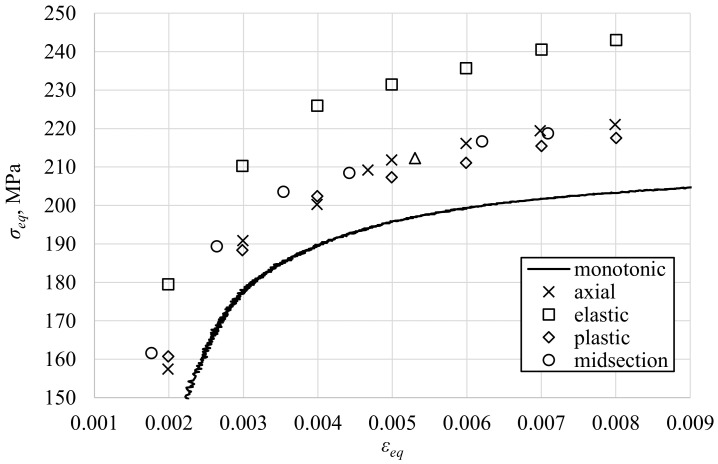
Cyclic midlife hardening analysis by application of different methods of shear stress determination.

Cyclic hardening is another phenomenon often investigated by researchers [52,53,54]. The interpretation of the experimental results is strongly related to the applied method of shear stress determination. To support this conclusion, the midlife Huber–Mises equivalent peak stresses from axial and torsional fatigue tests are presented in Figure 14 with a superimposed fragment of the monotonic tension stress–strain curve. For torsion, the equivalent stress was calculated based on the surface shear stress determined using the selected methods. A moderate cyclic axial hardening can be observed. If the elastic solution was applied for the shear stress determination, a higher amount of cyclic hardening under torsion could be found. This observation may be further interpreted as anisotropy in yield stress and hardening [55,56]. However, if the midsection or plastic solution were applied, the equivalent stresses under axial and torsional loading would be quite similar.

## 5. Summary

Based on the performed analyses of the experimental results of fatigue tests on thin-walled tubular specimens of PA38-T6 aluminum alloy, the following conclusions can be drawn:

1. None of the considered approximate solutions can determine the exact value of surface shear stress for tubular specimens under elastic–plastic cyclic torsional loading.
2. Using the midsection approach and purely plastic solution for high strain levels, it was found that values of surface shear stress were very close to the values obtained using the Chaboche nonlinear kinematic model, calibrated using the experimental data for the axial loading.
3. The largest differences between shear stress values calculated using the plasticity model and other methods were found for the low strain ranges, close to the cyclic yield limit.
4. When the ratio of inner and outer diameters of tubular specimens is close to 1, the effect of the nonuniform shear stress distribution through wall thickness is less pronounced.
5. It was confirmed that the recommendations of the ASTM standard can be successfully implemented to approximately determine the surface shear stress under torsion; however, if the applied loading levels cover the boundary between elastic and elastic–plastic deformation, it should be carefully considered which loading levels justify choosing a purely elastic or midsection approach.
6. The methods discussed are not valid for the full range of loadings. The selection of the approach should be based on the applied loading level and observed material response.
7. The calculated values of shear stress can have a strong impact on the fatigue life prediction, depending on the selected fatigue damage parameter. Thus, it is recommended to clearly indicate how the shear stress values are determined in a study when presenting the results of an experiment.

## Figures and Tables

**Table 1:** Chemical composition of PA38-T6 aluminum alloy according to EN 573-3 standard.

Element	Si	Fe	Cu	Mn	Mg	Cr	Zn	Ti	Other	Al
**Share (%)**	0.3–0.6	0.1–0.3	0.1	0.1	0.35–0.6	0.05	0.15	0.1	0.15	Balance

**Table 2:** Basic mechanical properties of PA38-T6 aluminum alloy determined experimentally.

*E*, GPa	$\sigma_{y02}$, MPa	$\sigma_{u}$, MPa	$\varepsilon_{\sigma_{u}}$, mm/mm	$\nu_{e}$, -	$K^{\prime }$, MPa	$n^{\prime }$, -
68.3	191.5	229.1	0.094	0.35	288.1	0.051

**Table 3:** Chaboche model material parameters determined for PA38-T6 aluminum alloy.

$C^{1}$, MPa	$C^{2}$, MPa	$C^{3}$, MPa	$\gamma^{1}$, -	$\gamma^{2}$, -	$\gamma^{3}$, -	*R*, MPa
$1.3723\times 10^{5}$	$5.3704\times 10^{4}$	$1.6667\times 10^{4}$	$7.8078\times 10^{3}$	$1.9759\times 10^{3}$	459.3	141.1

## References

[1] Tu S.T., Zhang X.C. (2016). Fatigue Crack Initiation Mechanisms. Ref. Modul. Mater. Sci. Mater. Eng..

[2] Rozumek D., Marciniak Z. (2012). The investigation of crack growth in specimens with rectangular cross-sections under out-of-phase bending and torsional loading. Int. J. Fatigue.

[3] Macek W. (2019). Fractal analysis of the bending-torsion fatigue fracture of aluminium alloy. Eng. Fail. Anal..

[4] Karolczuk A., Kluger K., Łagoda T. (2016). A correction in the algorithm of fatigue life calculation based on the critical plane approach. Int. J. Fatigue.

[5] Wei Z., Dong P. (2014). A generalized cycle counting criterion for arbitrary multi-axial fatigue loading conditions. J. Strain Anal. Eng. Des..

[6] Macek W., Łagoda T., Mucha N. (2018). Energy-based fatigue failure characteristics of materials under random bending loading in elastic-plastic range. Fatigue Fract. Eng. Mater. Struct..

[7] Krzyżak D., Kurek M., Łagoda T., Sówka D. (2014). Influence of changes of the bending plane position on the fatigue life. Mater. Werkst..

[8] Arora P., Gupta S.K., Bhasin V., Singh R., Sivaprasad S., Tarafder S. (2016). Testing and assessment of fatigue life prediction models for Indian PHWRs piping material under multi-axial load cycling. Int. J. Fatigue.

[9] Skibicki D., Pejkowski Ł., Stopel M. (2017). Finite element analysis of ventilation system fire damper dynamic time-history. Pol. Marit. Res..

[10] Bildhauer M., Fuchs M., Repin S. (2009). The elastic-plastic torsion problem: A posteriori error estimates for approximate solutions. Numer. Funct. Anal. Optim..

[11] Idone G., Maugeri A., Vitanza C. (2003). Variational inequalities and the elastic-plastic torsion problem. J. Optim. Theory Appl..

[12] Hout J.W., Benedict R.L. (1984). The Elastic-Plastic Torsion Problem: A Direct Boundary Approach. J. Struct. Mech..

[13] Annin B.D. (1965). Existence and uniqueness of the solution of the elastic-plastic torsion problem for a cylindrical bar of oval cross-section. J. Appl. Math. Mech..

[14] Caffarelli L.A., Friedman A. (1981). Unloading in the elastic-plastic torsion problem. J. Differ. Equations.

[15] Rubinstein R. (1977). On the elastic-plastic torsion problem. J. Eng. Math..

[16] Wu P.D., Van der Giessen E. (1991). Analysis of elastic-plastic torsion of circular bars at large strains. Arch. Appl. Mech..

[17] May I.M., Al-Shaarbaf I.A. (1989). Elasto-plastic analysis of torsion using a three-dimensional finite element model. Comput. Struct..

[18] Stout R.B., Hodge P.G. (1970). Elastic/plastic torsion of hollow cylinders. Int. J. Mech. Sci..

[19] Wagner W., Gruttmann F. (2001). Finite element analysis of Saint-Venant torsion problem with exact integration of the elastic-plastic constitutive equations. Comput. Methods Appl. Mech. Eng..

[20] Shoemaker E.M. (1973). Second order effects in the elastic-plastic torsion problem. Meccanica.

[21] Miller K., Chandler D.C. (1969). High Strain Torsion Fatigue of Solid and Tubular Specimens. Proc. Inst. Mech. Eng..

[22] Brown M.W. (1978). Torsional stresses in tubular specimens. J. Strain Anal. Eng. Des..

[23] Shamsaei N., Fatemi A. (2009). Effect of hardness on multiaxial fatigue behaviour and some simple approximations for steels. Fatigue Fract. Eng. Mater. Struct..

[24] Socie D.F. (1987). Multiaxial Fatigue Damage Models. J. Eng. Mater. Technol. Trans. ASME.

[25] Zhong B., Wang Y., Wei D., Zhang K., Wang J. (2018). Multiaxial fatigue life prediction for powder metallurgy superalloy FGH96 based on stress gradient effect. Int. J. Fatigue.

[26] Wu Z.R., Li X., Fang L., Song Y.D. (2018). Evaluation of multiaxial fatigue life prediction criteria for Ni-based superalloy GH4169. Proc. Inst. Mech. Eng. Part J Mech. Eng. Sci..

[27] Zhang J., Jiang Y. (2008). Constitutive modeling of cyclic plasticity deformation of a pure polycrystalline copper. Int. J. Plast..

[28] Zhang J., Jiang Y. (2005). An experimental investigation on cyclic plastic deformation and substructures of polycrystalline copper. Int. J. Plast..

[29] Sharifimehr S., Fatemi A. (2019). Fatigue analysis of ductile and brittle behaving steels under variable amplitude multiaxial loading. Fatigue Fract. Eng. Mater. Struct..

[30] McClaflin D., Fatemi A. (2004). Torsional deformation and fatigue of hardened steel including mean stress and stress gradient effects. Int. J. Fatigue.

[31] Dey R., Tarafder S., Sivaprasad S. (2019). Influence of proportional and non-proportional loading on deformation behaviour of austenitic stainless steel-macro and micro analysis. Theor. Appl. Fract. Mech..

[32] Gryguć A., Behravesh S., Shaha S., Jahed H., Wells M., Williams B., Su X. (2019). Multiaxial cyclic behaviour of extruded and forged AZ80 Mg alloy. Int. J. Fatigue.

[33] Han Q., Wang P., Lu Y. (2019). Low-cycle multiaxial fatigue behavior and life prediction of Q235B steel welded material. Int. J. Fatigue.

[34] Albinmousa J., Adinoyi M.J., Merah N. (2019). Multiaxial fatigue of extruded ZK60 magnesium alloy. Fatigue Fract. Eng. Mater. Struct..

[35] Ogawa F., Itoh T., Yamamoto T. (2018). Evaluation of multiaxial low cycle fatigue cracks in Sn-8Zn-3Bi solder under non-proportional loading. Int. J. Fatigue.

[36] Ting T.W. (1979). The repeated loading-unloading processes of elastic-plastic torsion of solid bars. Ann. Mat. Pura Appl..

[37] Bruhns O.T. (2003). Advanced Mechanics of Solids.

[38] Chaboche J.L. (1986). Time-independent constitutive theories for cyclic plasticity. Int. J. Plast..

[39] Chaboche J. (1989). Constitutive equations for cyclic plasticty and cyclic viscoplasticity. Int. J. Plast..

[40] Fatemi A., Socie D.F. (1988). A Critical Plane Approach To Multiaxial Fatigue Damage Including Out-of-Phase Loading. Fatigue Fract. Eng. Mater. Struct..

[41] Smith K., Watson P., Topper T. (1970). Stress-Strain Function for the Fatigue of Metals. J. Mater..

[42] Walat K., Kurek M., Ogonowski P., Łagoda T. (2012). The multiaxial random fatigue criteria based on strain and energy damage parameters on the critical plane for the low-cycle range. Int. J. Fatigue.

[43] Ince A., Glinka G. (2014). A generalized fatigue damage parameter for multiaxial fatigue life prediction under proportional and non-proportional loadings. Int. J. Fatigue.

[44] Garud Y.S. (1981). A New Approach to the Evaluation of Fatigue Under Multiaxial Loadings. J. Eng. Mater. Technol..

[45] Ellyin F., Golos K., Xia Z. (1991). In-Phase and Out-of-Phase Multiaxial Fatigue. J. Eng. Mater. Technol..

[46] Ellyin F. (1997). Fatigue Damage, Crack Growth and Life Prediction.

[47] Skibicki D., Pejkowski Ł. (2019). The relationship between additional non-proportional hardening coefficient and fatigue life. Int. J. Fatigue.

[48] Pejkowski Ł., Skibicki D. (2019). Stress-strain response and fatigue life of four metallic materials under asynchronous loadings: Experimental observations. Int. J. Fatigue.

[49] Karolczuk A., Skibicki D., Pejkowski Ł. (2019). Evaluation of the Fatemi-Socie damage parameter for the fatigue life calculation with application of the Chaboche plasticity model. Fatigue Fract. Eng. Mater. Struct..

[50] Li H., Lv F., Xiao Z., Liang X., Sang F., Li P. (2016). Low-cycle fatigue behavior of a cast Mg–Y–Nd–Zr alloy by T6 heat treatment. Mater. Sci. Eng. A.

[51] Wu D.L., Xuan F.Z., Guo S.J., Zhao P. (2016). Uniaxial mean stress relaxation of 9–12% Cr steel at high temperature: Experiments and viscoplastic constitutive modeling. Int. J. Plast..

[52] Li Y., Yu D., Li B., Chen X. (2019). Martensitic transformation of an austenitic stainless steel under non-proportional cyclic loading. Int. J. Fatigue.

[53] Molaei R., Fatemi A., Phan N. (2018). Significance of hot isostatic pressing (HIP) on multiaxial deformation and fatigue behaviors of additive manufactured Ti-6Al-4V including build orientation and surface roughness effects. Int. J. Fatigue.

[54] Fu Y.S.S., Chen S.S.X. (2018). Torsional fatigue with axial constant stress of oligo-crystalline 316L stainless steel thin wire. Fatigue Fract. Eng. Mater. Struct..

[55] Lin H., Nayeb-Hashemi H., Pelloux R.M. (1992). Constitutive relations and fatigue life prediction for anisotropic Al-6061-T6 rods under biaxial proportional loadings. Int. J. Fatigue.

[56] Zhang J., Yu Q., Jiang Y., Li Q. (2011). An experimental study of cyclic deformation of extruded AZ61A magnesium alloy. Int. J. Plast..

[57] (2002). Standard Practice for Strain-Controlled Axial-Torsional Fatigue Testing with Thin-Walled Tubular Specimens.

